# Relationship between Neonatal MRI Findings and Emotional/Behavioral Evaluation in Early Childhood for Extremely Low-Birth-Weight Infants

**DOI:** 10.3390/jcm11030772

**Published:** 2022-01-31

**Authors:** Akinobu Taniguchi, Masahiro Hayakawa, Erina Kataoka, Naozumi Fujishiro, Yoshiaki Sato

**Affiliations:** 1 Division of Neonatology, Center for Maternal-Neonatal Care, Nagoya University Hospital, Nagoya 466-8560, Japan; masahaya@med.nagoya-u.ac.jp (M.H.); yoshiaki@med.nagoya-u.ac.jp (Y.S.); 2 Department of Pediatrics, Anjo Kosei Hospital, Anjo 446-8602, Japan; kataokaerina0903@gmail.com; 3 Department of Pediatrics, Japanese Red Cross Aichi Medical Center Nagoya Daiichi Hospital, Nagoya 453-8511, Japan; naozumifujishiro@yahoo.co.jp

**Keywords:** low-birth-weight infants, neonatal MRI, Strength and Difficulties Questionnaire (SDQ), Global Brain Abnormality Score, Developmental Quotient

## Abstract

The aim of this study is to investigate whether it is possible to detect future behavioral and emotional problems in extremely low-birth-weight infants by evaluating the neonatal head magnetic resonance imaging (MRI) using a scoring system. This study included 62 extremely low-birth-weight infants born between April 2015 and March 2017 and those who had undergone MRI at 36 to 42 weeks of gestation. These subjects were administered with the Strength and Difficulties Questionnaire (SDQ) at age 4–5, and the patients who responded to the questionnaire were included in the study. A positive correlation was observed between the Global Brain Abnormality Score and Total Difficulties Score of the SDQ (r = 0.26, *p* = 0.038). However, no significant difference was observed between the median Global Brain Abnormality Score of the normal and borderline-range group and the Total Difficulties Score of the clinical-range group (*p* = 0.51). This study demonstrated the relationship between the MRI findings in the newborn period and the emotional and behavioral problems in early childhood, but it is not clinically useful as a predictive marker.

## 1. Introduction

It is widely known that very low-birth-weight infants have delayed motor and cognitive functions during their development [1]. Moreover, it has been reported that preterm infants often have behavioral and emotional problems in early childhood and school age [2,3,4,5]. The behavioral and emotional problems of very low-birth-weight infants can interfere with their daily lives and cause problems for them and their parents.

A previous study showed a significant improvement in behaviors with the help of mental health promotion and prevention programs [6]. If we can identify the signs of behavioral and emotional problems early, it is possible to reduce these problems. However, to date, no indicators for predicting behavioral and emotional problems in early childhood have been established.

A previous study reported that there was association between neonatal MRI and cognition, motor skills at 2 years of age [7]. It was also reported that adolescents with behavioral and emotional difficulties tend to have a lower gray matter volume in the right orbitofrontal cortex based on the results of the magnetic resonance imaging (MRI) [8]. Another study showed that the behavioral problems of children were associated with low cognitive performance, developmental delay, hospitalizations of the child, young maternal age, and poor maternal mental well-being, and children born very preterm were at a higher risk of behavioral problems compared with children born full-term [5]. These findings suggest that behavioral and emotional difficulties may be due not only to environment factors and cognitive functioning but also to the fact that the child was born prematurely.

The aim of this study is to investigate whether it is possible to detect future behavioral and emotional problems in extremely low-birth-weight infants by evaluating neonatal head MRI using a scoring system (Kidokoro score).

## 2. Materials and Methods

### 2.1. Study Design and Participants

This study was a multicenter study, and the study population included extremely low-birth-weight infants (with a birth weight of less than 1000 g) born between April 2015 and March 2017 and those who had undergone MRI at 36 to 42 weeks of gestation at Nagoya University Hospital, Aichi, Japan, and joint research facilities (Anjo Kosei Hospital, Japanese Red Cross Aichi Medical Center Nagoya Daiichi Hospital, Konan Kosei Hospital, Ogaki Municipal Hospital, Okazaki City Hospital, Tosei General Hospital, Toyota Memorial Hospital). The subjects were administered the Strength and Difficulties Questionnaire (SDQ) at age 4–5, and the patients who responded to the questionnaire were included in the study. Perinatal clinical data in the neonatal care unit and the results of developmental test were retrospectively investigated.

### 2.2. Brain Injury Assessment of MRI

The MRI of the neonatal brain at around term-equivalent age was performed at the facility where each child was born. The MRI was undertaken by using a 3T system without sedation and included anatomic images obtained with an axial magnetization-prepared rapid acquisition of gradient echo T1-weighted sequence and a turbo spin-echo T2-weighted sequence. The neonatal MRI scans were reviewed by two experienced neonatologists. They were blinded to any clinical characteristics or outcome data of the children except for their post-menstrual age (PMA) at the time of scanning. If there was a difference between the two neonatologists’ readings, the score was decided after consultation between them. The MRI examinations performed at 36–42 weeks of PMA were included. The neonatal MRI scans were assessed using a standardized scoring system (Kidokoro score) to assess abnormal brain metrics and the presence and severity of abnormalities in the cerebral white matter, cortical and deep gray matter, and cerebellum. The sum of these scores leads to a Global Brain Abnormality Score (GBAS), which can be further classified as normal (0–3), mildly abnormal (4–7), moderately abnormal (8–11), and severely abnormal (12–) [9].

### 2.3. Developmental Testing

In patients who had undergone a developmental examination, the results of the Kyoto Scale of Psychological Development 2001 at modified 1.6 and 3 years of age were also retrospectively investigated. The Kyoto Scale of Psychological Development is a standardized and validated developmental test available at all centers participating in the follow-up study of the Neonatal Research Network, Japan, but has not been published or standardized in English [10]. It is also an individualized face-to-face test used to assess the child’s development in the following three areas: Postural–Motor (P–M; fine and gross motor functions), Cognitive–Adaptive (C–A; non-verbal reasoning or visuospatial perceptions assessed using materials), and Language–Social (L–S; interpersonal relationships, socialization, and verbal abilities). Each score for these three areas and the sum of the scores were converted for each developmental age and overall developmental age. The Developmental Quotient (DQ) was calculated by dividing the developmental age by the corrected age for prematurity and then multiplying the quotient by 100. The corrected age was adjusted using the estimated date of birth instead of the chronological date of birth. In the Japanese protocol for the follow-up for very low-birth-weight infants, developmental function was classified as “delayed” when the overall DQ was <70, “subnormal” when the DQ was 70–84, and “normal” when the DQ was ≥85 [10]. A previous study showed that the developmental characteristics on the Kyoto Scale of Psychological Development were well correlated with those on the Bayley III [11]. All developmental testing in the study was carried out by psychotherapists based at the participating institutions.

### 2.4. Evaluation of Behavioral and Emotional Problems

The subjects were administered the SDQ at age 4–5. The SDQ was developed by Goodman and colleagues in the UK as an open access, downloadable screening tool, available as a self-report, parent/caregiver-report, and teacher-report version [12]. The SDQ was designed as a brief rating instrument to assess the behaviors of 4- to 16-year-old children. In the present study, the Japanese version of parent/caregiver-report SDQ was used as a behavioral screening questionnaire for children. The SDQ items are divided into five scales with five items each: Emotional Symptoms Scale, Conduct Problems Scale, Hyperactivity Scale, Peer Problems Scale, and Prosocial Scale. Each item can be marked as “not true,” “somewhat true,” or “certainly true.” The score for each of the five scales is generated by summing the scores for the five items that make up that scale, thereby generating a scale score ranging from 0 to 10. The scores for hyperactivity, emotional symptoms, conduct problems, and peer problems can be summed to generate a Total Difficulties Score ranging from 0 to 40. In the present study, the parent/caregiver-report version of SDQ was used to obtain responses from parents. The questionnaires were sent to the parents by mail, and the responses were collected by mail. In this study, we used the SDQ standard values for preschool children (4–5 years old) in Japan and defined the Total Difficulties Score of 0–11 as the normal range, 12–13 as the borderline range, and 14–40 as the clinical range for boys as well as the Total Difficulties Score of 0–9 as normal range, 10–12 as borderline range, and 13–40 as clinical range for girls (clinical range: 90–100% tiles of the cohort population, borderline range: 80–90% tiles of the cohort population, normal range: 0–80% tiles of the cohort population) [13].

### 2.5. Outcomes

The primary outcome was defined as the association between the Total Difficulties Score and GBAS. The secondary outcomes were defined as the association between the Total Difficulties Score and DQ at modified 1.5 or 3 years of age and other demographic characteristics.

### 2.6. Statistics Analysis

JMP^®^ Pro 15 (SAS Institute Inc., Cary, NC, USA) was used for all analyses. Correlation analysis was used to analyze the correlation between the two types of quantitative data. Student’s *t*-test was used to compare the values between the two groups. *p*-Values of <0.05 were considered statistically significant.

### 2.7. Ethical Issues

Informed consent to participate in this study was obtained in writing. Since the subjects of our investigation are children, consent was obtained from their parents. The children whose consent could not be obtained from their parents or guardian were excluded. This study was approved by the local ethical committee at Nagoya University Hospital (No. 2020-0241, approved 11 September 2020).

## 3. Results

### 3.1. Characteristics of the Study Participants

Overall, 143 extremely low-birth-weight infants who had undergone head MRI at 36 to 42 weeks of gestation and 62 patients were included in the study, excluding 8 patients who had inadequate images and 73 patients who did not respond to the questionnaire. Sixty-one (98%) patients had undergone developmental testing at modified 1.6 years of age using the Kyoto Scale of Psychological Development 2001, and fifty-seven (92%) had undergone developmental testing using the Kyoto Scale of Psychological Development 2001 at 3 years old. Table 1 shows the perinatal demographic characteristics of the subjects.

### 3.2. Primary Outcome

**Figure 1:**
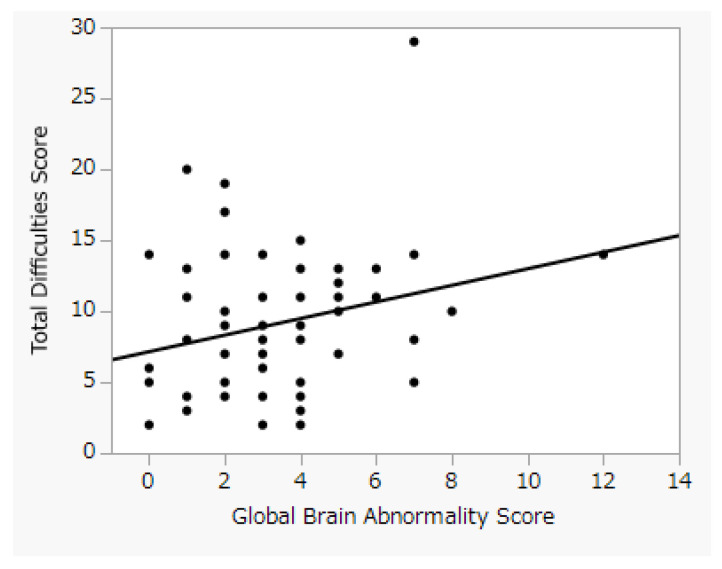
Relationship between Global Brain Abnormality Score and Total Difficulties Score. The correlation coefficient between GBAS and Total Difficulties Score was 0.26 (*p* = 0.038).

The mean value and median value of the Total Difficulties Score among the subjects in this study were 9.10 ± 4.89 and 8 (5–11.25), respectively. A positive correlation was observed between the GBAS and Total Difficulties Score (r = 0.26, *p* = 0.038) (Figure 1). However, the median GBAS between the normal- and borderline-range group and the Total Difficulties Score of the clinical-range group were 3 (2–4) and 2.5 (1.75–7), respectively, and no significant difference was observed between the two groups (*p* = 0.51).

### 3.3. Secondary Outcomes

A significant correlation was observed between the GBAS and DQ at modified 1.5 years of age, but no significant correlation was observed between the GBAS and DQ at 3 years old (Table 2). Moreover, no significant correlation was observed between the DQ and Total Difficulties Score (Table 3). Gestational age, birth weight, maternal age at delivery, and the other demographic characteristics in Table 1 were not related to the Total Difficulties Score.

## 4. Discussion

This study showed the correlation between the GBAS and Total Difficulties Score. However, no significant difference was observed in the GBAS between the normal- and borderline-range group and the Total Difficulties Score of the clinical-range group, which was not clinically significant. No significant correlation was observed between the DQ or demographic characteristics and the Total Difficulties Score. Due to the relatively small number of subjects in this study, it cannot be ruled out that a small number of outliers may have affected the correlation.

The mean value of the Total Difficulties Score among the subjects in this study was 9.10 ± 4.89, which was higher than the mean value of 7.47 ± 4.67 in a Japanese study of children at age 4–5 [13]. This finding is consistent with that of previous studies that have shown that preterm infants are more likely to develop problems.

The present study demonstrated an association between the MRI findings in the neonatal period and emotional and behavioral problems in early childhood. The association between the head MRI at modified 36 to 42 weeks of age and SDQ results at 4–5 years of age suggests that perinatal problems may be related to future emotional and behavioral problems. In the present study, we used the Kidokoro score to evaluate the head MRI in the neonatal period, which can be easily evaluated by anyone without special analysis software and may be clinically accessible. Moreover, the Kidokoro score was not clinically useful as a predictive marker of behavioral and emotional problems in early childhood. However, we hypothesized that combining the Kidokoro score with other clinical data to create a clinically useful scoring system may become an issue in future research. At present, it is difficult to predict behavioral and emotional problems based only on neonatal MRI and developmental tests in early childhood. Therefore, it is necessary to have a follow-up system that focuses on behavioral and emotional problems in the early childhood of very low-birth-weight infants.

It is difficult to refer to structural brain causes as to why the association was found in this study, but a previous study suggests that childhood conduct problems are related to reduced fiber-specific microstructures within the white matter fiber pathway [14]. Common behavioral disorders in childhood and adolescence, such as oppositional defiant disorder and conduct disorder, are associated with brain abnormalities [15].

The results of developmental testing at age 3 showed no association with emotional or behavioral problems in early childhood. Previous reports showed that cognitive function was associated with behavioral problems [5], but in the present study, we did not find a significant association. If the results of the developmental tests alone are used as indicators, it may not be possible to identify emotional and behavioral problems in the follow-up process of extremely low birth weight infants.

One of the limitations of the present study is the exclusion of patients who did not undergo a head MRI at 36–42 weeks of PMA. Because the GBAS assessment study included patients who had undergone head MRI at 36–42 weeks of PMA, cases in which a head MRI could not be obtained during that period were excluded. It is possible that patients with poor general condition who could not undergo head MRI at this time were excluded from the study. It is therefore possible that the cases included in the study were biased towards those in relatively good condition. Another limiting factor was that we only conducted evaluations from the parents and not from the children themselves or their teachers. How the children responded to the questions is also an important indicator, but in this case, we were not able to examine the evaluation from the children themselves since we were targeting children as young as 4–5 years old.

Another limitation is that about half of the responses in the SDQ survey were not obtained. We were unable to get a higher response rate because some cases were transferred or dropped out of the hospital. However, as a multicenter study, we were able to include cases of transfers between the collaborating institutions, which can be considered a strength of this study. We also cannot rule out the possibility that parents of patients with severe complications refrained from cooperating with the study. It is therefore possible that the cases included in the study were biased towards those in relatively good condition.

We did not collect data on the maternal socioeconomic status, educational status, and mental health, which have been previously shown to influence neurodevelopmental outcomes of ELBW infants. With regard to the mother’s age, which is associated with other factors, the present study did not find an association with behavioral problems.

In this study, we were able to confirm the relationship between the MRI findings in the newborn period and emotional and behavioral problems in early childhood. Perinatal management that reduces abnormalities in the head MRI in the neonatal period may improve future emotional and behavioral problems. Future research is needed to identify clinically useful predictive markers of behavioral and emotional problems in early childhood. In the current situation, it is considered important to have a follow-up system that focuses on the behavioral and emotional problems in early childhood of very low-birth-weight infants.

## 5. Conclusions

This study demonstrated the relationship between MRI findings in the newborn period and emotional and behavioral problems in early childhood. However, the GBAS was not clinically useful as a predictive marker of behavioral and emotional problems in early childhood.

## Figures and Tables

**Table 1:** The perinatal demographic characteristics.

	Overall (*n* = 62)	GBAS Normal (*n* = 36)	GBAS Mild (*n* = 24)	GBAS Moderate- Severe (*n* = 2)
Females/males	27/35	19/17	7/17	1/1
Gestational age (weeks/days) ^†^	27/3 (25/2–28/5)	27/3 (25/1–28/5)	25/3 (24/2–27/5)	27/6 (25/5–30/6)
Birth weight (g) ^†^	827.5 (676.5–935.75)	880 (732–955)	707 (555.25–912.75)	720 (544–896)
Apgar score (5 min) ^†^	6 (3–7)	6 (5–7.75)	4 (2–7)	4.5 (2–7)
Singleton	51 (82%)	30 (83%)	19 (79%)	2 (100%)
Maternal age at delivery ^†^	33 (28–36.25)	34 (28–37)	29 (27.25–35.75)	38 (33–43)
Cesarean delivery	53 (85%)	32 (89%)	21 (88%)	2 (100%)
Pre-labor rupture of membranes	21 (34%)	15 (42%)	6 (25%)	0 (0%)
IVH (≥grade 3)	4 (6%)	1 (3%)	1 (4%)	2 (100%)
PVL	2 (3%)	1 (3%)	1 (4%)	0 (0%)
CP	4 (6%)	1 (3%)	2 (8%)	1 (50%)
ROP treatment	19 (31%)	11 (31%)	7 (29%)	1 (50%)
BPD	39 (63%)	21 (62%)	17 (71%)	1 (50%)
NEC	0 (0%)	0 (0%)	0 (0%)	0 (0%)
Sepsis	4 (6%)	2 (6%)	2 (8%)	0 (0%)

^†^, median (IQR); GBAS, Global Brain Abnormality Score; IVH, intraventricular hemorrhage; PVL, periventricular leukomalacia; CP, cerebral palsy; ROP, retinopathy of prematurity; BPD, bronchopulmonary dysplasia; NEC, necrotizing enterocolitis.

**Table 2:** Relationship between Global Brain Abnormality Score and developmental test results.

	DQ at Modified 1.5 Years of Age			DQ at 3 Years of Age		
	C–A	L–S	P–M	C–A	L–S	P–M
GBAS	r = −0.30 (*p* = 0.021)	r = −0.33 (*p* = 0.001)	r = −0.37 (*p* = 0.004)	r = −0.02 (*p* = 0.87)	r = −0.05 (*p* = 0.72)	r = −0.14 (*p* = 0.30)

DQ, Developmental Quotient; C–A, Cognitive–Adaptive; L–S, Language–Social; P–M, Posture-Motor; GBAS, Global Brain Abnormality Score.

**Table 3:** Relationship between developmental test results and Total Difficulties Score.

		Total Difficulties Score
DQ at modified 1.5 years of age	C–A	r = −0.23 (*p* = 0.07)
	L–S	r = −0.08 (*p* = 0.52)
	P–M	r = 0.00 (*p* = 0.98)
DQ at 3 years of age	C–A	r = −0.17 (*p* = 0.21)
	L–S	r = 0.05 (*p* = 0.74)
	P–M	r = −0.22 (*p* = 0.10)

DQ, Developmental Quotient; C–A, Cognitive–Adaptive; L–S, Language–Social; P–M, Posture-Motor.

## Data Availability

The data presented in this study are available on request from the corresponding author. The data are not publicly available due to privacy.
